# Removal of Urinary Calculi from Right Lumbar and Pubic Regions, by Incision

**Published:** 1858-06

**Authors:** J. W. Wyman

**Affiliations:** Beaufort District, So. Ca.


*Removal of Urinary Calculi from Right Lumbar and Pubic Regions, by Excision.* By J. W. WYMAN, M. D., of Beaufort District, So. Ca.

An interesting case of urinary calculi came under my charge as long ago as 1836. The victim of this dire scourge was a man of strong will and endurance, and could unflinchingly have been a martyr to any cause, to which his reason assented as just. He was then nearly fifty years of age, and for more than twenty years he had a history of suffering to relate which could not fail to interest the sympathy of humanity. Beginning while yet a young man, persecuting its victim for years, with only an occasional respite, and that, too, when the sufferer, by his ample means, had availed himself of medical advice and aid, and visited the several "watering places" of repute for such "ailments," it was at once set down as having its "play and affinities" upon a system of the constitutional diathesis.

Periodically, though at irregular intervals, numerous small calculi of different kinds, as far as my limited means of analysis enabled me to ascertain. were voided with the urine, after means had been used to allay constitutional and local irritation. Retention of urine from mechanical irritation of the bladder and neck was the most apparent proximate cause of suffering; but through reflex action, the stomach and even the nervous centres were profoundly implicated during the paroxysms.—When these subsided so much relief followed that I at once concluded there could not be in the bladder any large calculi, as there remained no marked symptoms of such a condition.

The local treatment which, during these intermissions of suffering, I concluded to be best adapted to meet the exigencies of the case, was to enlarge as much as possible the urethra, and thereby diminish its irritability and susceptibility to the influence of these mechanical causes of pain. I put my patient upon the daily use of regulated sizes of gum elastic bougies, which proved to be of great service.

The deposits showed that the oxylate of lime was the predominant compound, though mixed with these at times was a considerable discharge of the red sediment of uric acid, and some few of the triple phosphates, according to the tests of Golding Bird; and the appearance of the latter, I believe, is most often found associated with a calculus in the bladder, or some organic mischief of that organ or the kidneys.

Internally I prescribed the nitro-hydrochloric acid in solution to meet the indication of the oxylate; occasionally suspending its use and administering the well-known remedy, uva ursi and soda, the pipsissewa (Pyrola umbellata) and other kindred remedies, restricting the regimen to those articles of food most easily digested, and I ordered the clothing to be regulated so as to maintain a uniform temperature of the skin, and exercise to be taken in the open air, when the weather was suitable, so as to furnish the system with a full supply of oxygen.

The kidneys I regard as the emunctories of the devitalized constituents and compounds of the body, no longer of any service; the drainers of deleterious and useless fluids, and debris of secondary assimilation; and though the laws of vitality ever seem opposed to those of chemistry, in nutritive assimilation, and wonderfully exert their influence for the preservation and even restoration of the body, still when certain nonvitalized elements come together, whether in or out of the body, chemistry asserts its right to make up such compounds as come under the range of its laws of affinity. It seems peculiarly active in bringing its laws into operation among the constituents, which make their way through the circulation to the kidneys; and when favored by subjects of sedentary habits, ("free livers") impaired digestions and defective cutaneous functions, there is an assemblage of non-vitalized elements centred in the system which take on their play of affinities and fasten their results upon the small joints of the extremities, where the circulation is sluggish, and apply the rack of gout; or on the larger ones institute the tortues of rheumatism; or take their meandering course to the kidneys, and settle down into the no less dreaded form of calculi. These few incidental remarks are thrown out to show the probable causes of calculi, and how closely allied this disease is with other painful affections, which as if "to add injury to insult," sometimes combine their forces in the same subject.

Continuing my treatment for some little time, my patient, a man "of the strictest sect" as respected "his manner of life," for he had long been a sufferer, and had learned from experience the best regimen for his condition, enjoyed an interregnum of suffering for upwards of two years, and began to console himself with the idea that he was well nigh a sound man, when I was summoned to see him again suffering under a retention of urine. His general symptoms were those which are consequent upon depressed nervous energy, and resembled those of persons laboring under hypochondriasis, or severe dyspepsia, in addition to those of retention.—He wore an anxious look, and had a bounding pulse. Venesection and the warm bath were followed with a discharge of urine, preceded by a copious flow of mucus and pus, and perhaps some small calculi. Naturally erect in gait, I had recently observed in the patient some inclination of body to the right and forward, which change of posture he referred to "the small of his back." On examination of the region over the right kidney, I found a deep fluctuation, caused evidently by a large sac containing pus. I informed him of this circumstance, and expressed my belief that I had found the fountain from which flowed the pus that he had been discharging from his bladder. Stimulating poultices were ordered to be applied to the part, in order, if possible, to invite the contents of the sac to the surface, so that a surgical operation would be less painful and hazardous, for I had determined to relieve the burdened ureters from the labor of discharging so large a quantity of pus. In a few days there were slight indications of a swelling and redness; and fearing, if the operation were deferred much longer, that the sac might rupture internally, I determined to operate at once, stating that the case was anomalous to me, and the result of the operation, though the only alternative, doubtful; that in other regions and structures the same indications called for the surgical instrument; that though no doubt existed in my mind that the sac directly communicated with the kidney, still an operation might be the means of restoring him to tolerable health, and that deferred much longer, the lamp of life must be extinguished.

Accordingly a free incision was made about two inches from the spine, a little below the last false rib; this was followed by a full pint of pus, strongly impregnated with the peculiar aroma of urine, and from pressure, pure urine, came away with pus. The flow was stopped, and the orifice filled with a tent. On removing the tent the next day, more pus was voided, and in gently carrying a tent down to the bottom of the cavity, the probe came in contact with a hard substance, which I decided to be a calculus, the irritation of which was the cause of this large collection of pus, and the general symptoms of depressed nervous energy.—The calculus, after extraction, was found to be an inch and a quarter long, somewhat irregularly oblong, dumb-bell shaped, with small, sharp, rough elevations, and of a brownish color, answering to the description of of mulberry calculus, the oxylate of lime.

This operation was performed in October, 1838, and up to 1845, the patient's health was generally good, he being able to attend to his business, and represent the parish as senator for two terms. The last year of his public service unfortunately exposed him to the measles, and kindled up anew the latent remains of his old diathesis. In May, 1846, a small tumor made its appearance on the abdomen, about two inches above the pubis, an inch or so to the right of the median line, and put on the character of a boil, which, on puncturing, proved to contain a calculus. This had left its bed in the right kidney, made its way by the old cavity, until, impeded further by the cicatrix, it passed round just above the ilium, and following the course of the oblique muscle, made a lodgment, from which it was with some difficulty extracted, in consequence of the fibres of the structure being insinuated into the rough elevations of the stone.

By tonics and anti.lithics, health was measurably restored, and my patient shared, at the declining age of three-score years, a fair amount of health. The curtain of death dropped at the age of sixty-two.

This was a remarkable case of cheerfulness and suffering blended, and of a life prolonged under the pressure of great bodily infirmity through nearly two-thirds of its journey.—*Charleston Med. Journal & Review.*

